# Mature Testicular Teratoma with a Focus of Embryonal Carcinoma: A Case Report and Review of Literature

**DOI:** 10.7759/cureus.2329

**Published:** 2018-03-15

**Authors:** Waliul Chowdhury, Muhammad Uzair Lodhi, Intekhab Askari Syed, Umar Rahim, Mustafa Rahim

**Affiliations:** 1 Medical Student, Department of Medicine, Raleigh General Hospital, Beckley, Wv; 2 Pre-Medical Student, Department of Sciences, Queens University of Charlotte, Nc; 3 Assistant Clinical Professor of Internal Medicine, West Virginia University School of Medicine

**Keywords:** testicular teratoma, embryonal carcinoma, testicular cancer

## Abstract

We present a 37-year-old male patient with a mature teratoma of the right testicle with a focus of embryonal carcinoma. This patient’s tumor metastasized radically to the retroperitoneum, right adrenal gland, bilateral lungs, and liver. A metastatic teratoma with embryonal carcinoma in males is a very rare case. We will describe our patient’s case, diagnostic workup, and management in detail, in addition to reviewing the related literature.

## Introduction

Testicular cancers are rare and are represent roughly 1% of all malignant tumors in males. The majority of testicular cancers are germ cell tumors, which is one of the most common causes of cancer-related deaths in males between the ages of 15 to 35. The lungs are the most common sites of metastasis. Typical components of a teratoma include cystic lesions lined by structures like the respiratory epithelium, smooth muscle, striated muscle, cartilage, or lymphoid tissues [1]. We present a 37-year-old male with pain and swelling in the right groin. He was diagnosed with a mature testicular teratoma with a small focus of embryonal carcinoma. His findings on imaging were very severe and out of proportion with his clinical presentation. In addition, the tumor was barely visible on gross inspection upon orchiectomy.

## Case presentation

History and physical examination

A 37-year-old Caucasian male presented with intermittent pain in the right groin. The pain had persisted for months. The pain was radiating to his upper abdomen. He denied any recent weight loss or trauma. The patient denied any past medical history or previous surgeries. His father had testicular cancer. The patient smoked one pack of cigarettes per day. He denied any alcohol or illicit drug use. He worked for a logging company, and initially assumed that his pain was associated with heavy lifting. He did not mention any allergy to medications. His blood pressure upon presentation was 151/86. The patient was in slight distress due to pain in the right groin. Tenderness in the right inguinal area was noted on physical examination. The right testicle was larger in size compared to the left testicle. No discharge was noted.

Hospital course

Diagnostic Workup

His complete blood count is shown in Table 1. The metabolic panel is shown in Table 2. Urinalysis results are shown in Table 3. Tumor markers are shown in Table 4. 

**Table 1:** Complete Blood Count

Test	Result
White blood cells	7.6 x 10^3 ^per mm^3^
Red blood cells	5.1 x 10^6 ^per mm^3^
Hemoglobin	15.4 g/dL
Hematocrit	46.2%
Mean corpuscular volume	91 µm^3^
Mean corpuscular hemoglobin concentration	33 %
Red cell distribution width	13.3 %
Platelet count	258 x 10^3 ^per mm^3^
Granulocyte percentage	68.3 %
Absolute neutrophil count	5.2 x 10^3 ^per mm^3^
Lymphocyte percentage	18.2 %
Monocyte percentage	10.5 %

**Table 2:** Metabolic Panel

Test	Result
Chloride	103 mmol/L
Carbon dioxide	26 mmol/L
Anion gap	13 mmol/L
Blood urea nitrogen	8 mg/dL
Creatinine	0.7 mg/dL
Albumin	3.4 g/dL
Calcium	9.1 mg/dL
Estimated glomerular filtration rate	> 60
Phosphate	4.5 mg/dL
Magnesium	2 mg/dL
Prothrombin time	10.1 seconds
International normalized ratio	0.99
Partial thromboplastin time	24 seconds
Total protein	8 g/dL
Albumin	3.9 g/dL
Globulin	4.1 g/dL
Albumin/globulin ratio	1
Total bilirubin	0.5 mg/dL
Aspartate aminotransferase	24 units/L
Alanine transaminase	18 units/L
Total alkaline phosphatase	49 units/L
Lipase	104 units/L

**Table 3:** Urinalysis Result

Test	Result	Reference
Color	Amber	Yellow
Appearance	Clear	Clear
Nitrate	Negative	Negative
Leukocyte esterase	Negative	Negative

**Table 4:** Tumor Markers

Test	Result
Beta-human chorionic gonadotropin	> 20,000 mIU/mL
Lactate dehydrogenase	757 units/L
Alpha-fetoprotein	126.9 ng/mL

**Figure 1:**
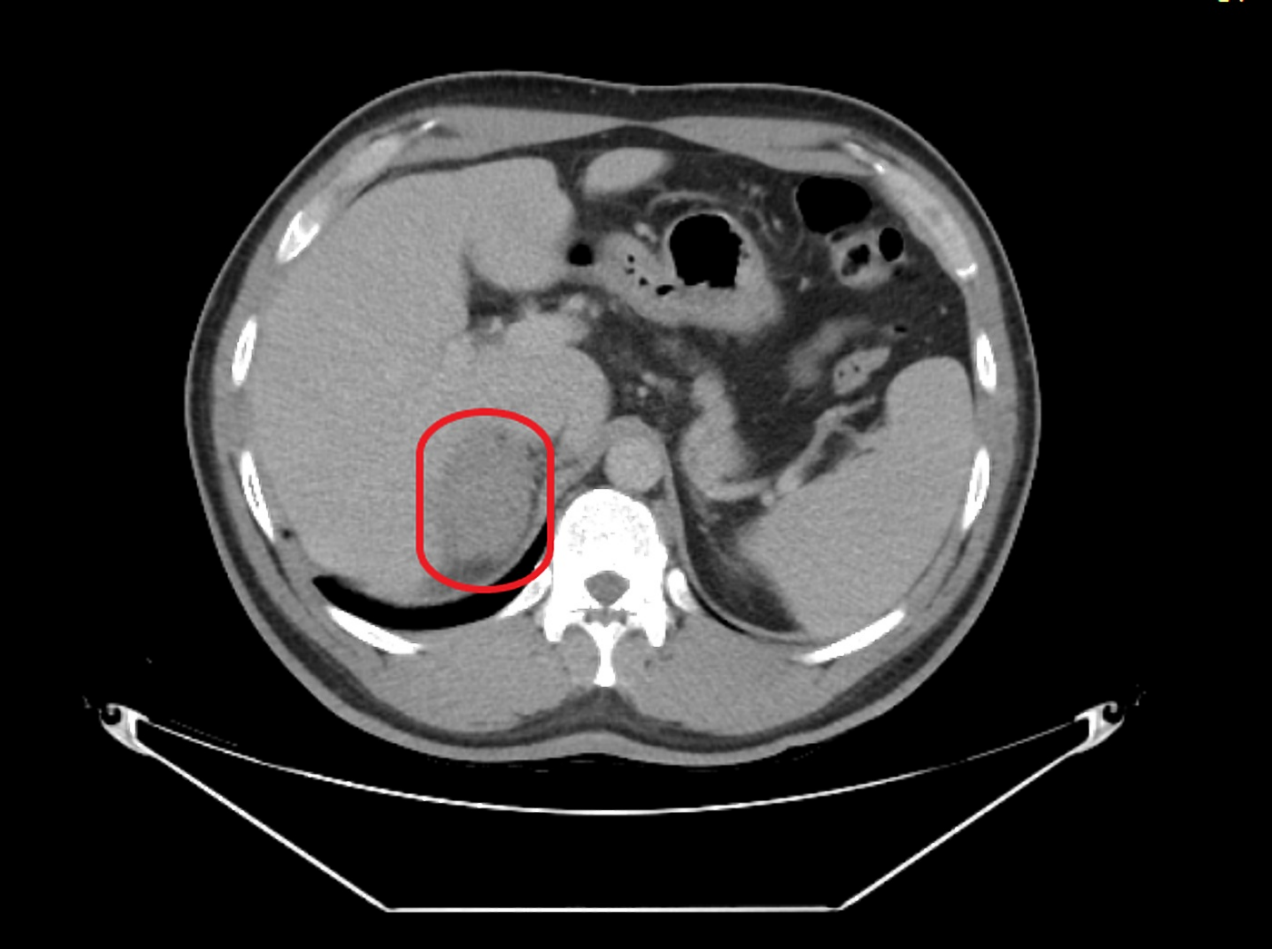
Abdominal computed tomography (CT) scan showing a metastatic lesion in the liver (red circle)

**Figure 2:**
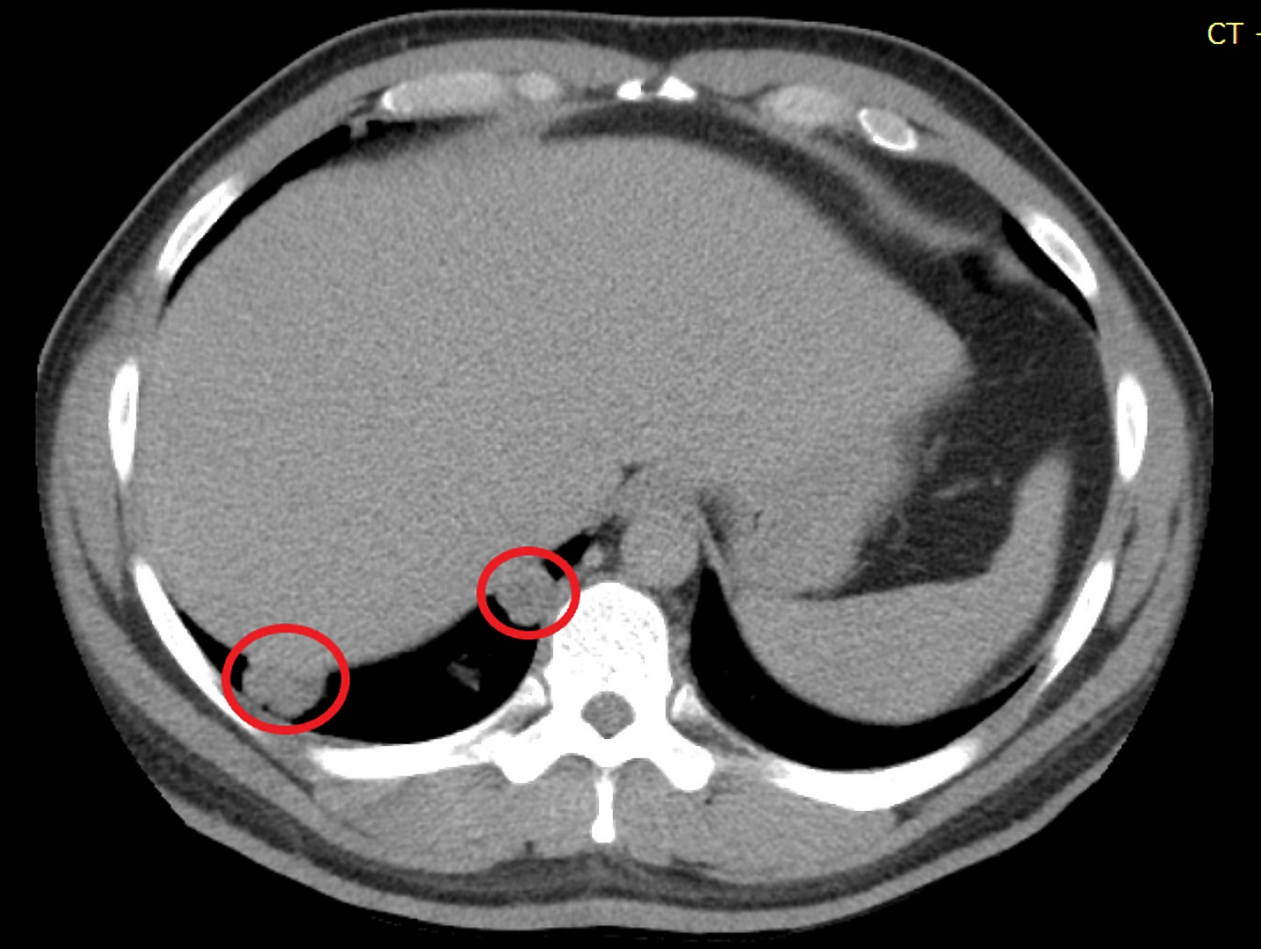
Abdominal computed tomography (CT) scan showing multiple metastatic lesions in the liver (red circles)

**Figure 3:**
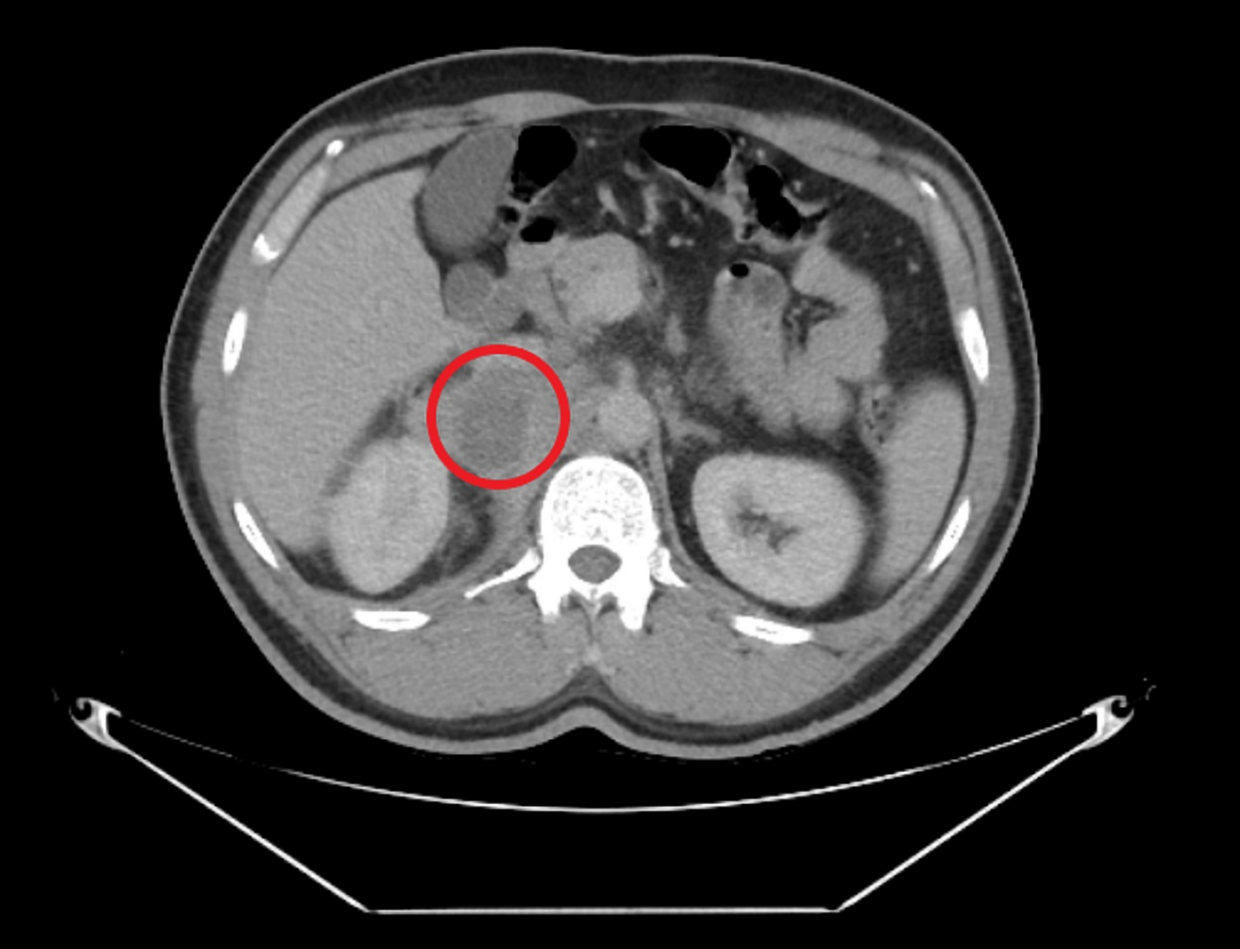
Pelvic computed tomography (CT) scan showing a mass lesion on the right adrenal gland (red circle)

**Figure 4:**
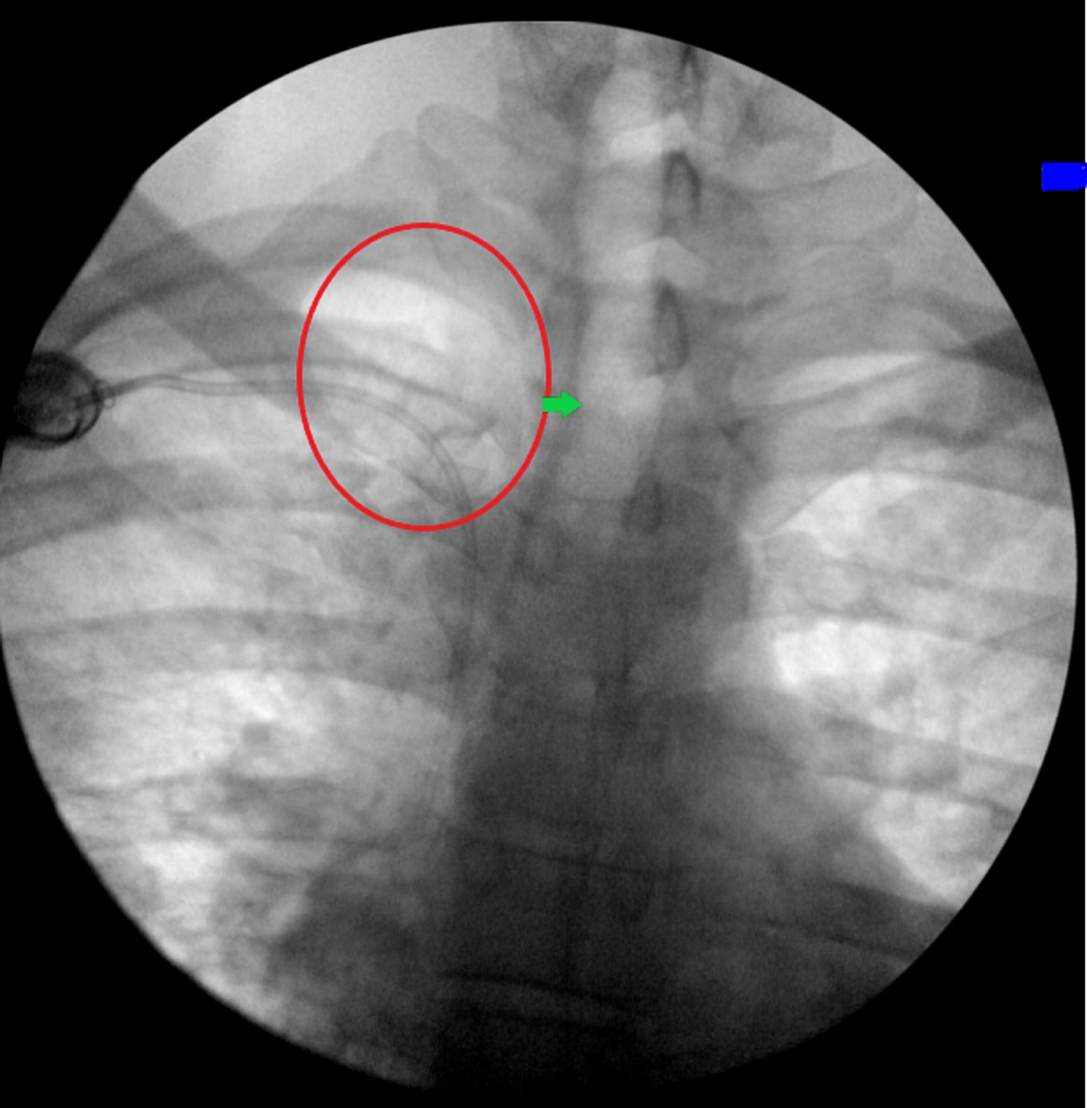
Abdominal computed tomography (CT) scan showing a retroperitoneal mass (red circle) and displacement of the aorta (green arrow)

**Figure 5:**
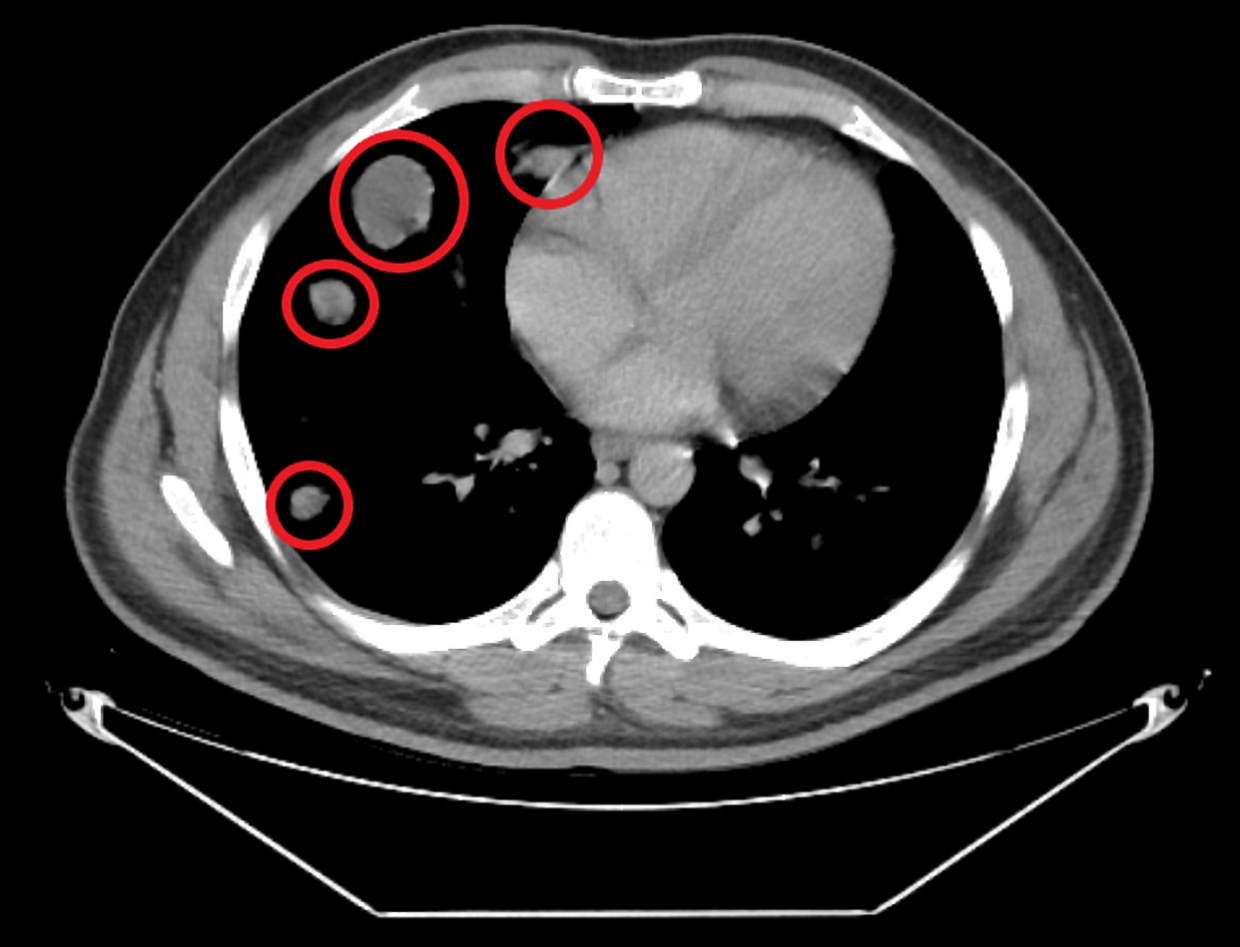
Chest computed tomography (CT) scan showing multiple metastatic pulmonary lesions (red circles) on the right side

**Figure 6:**
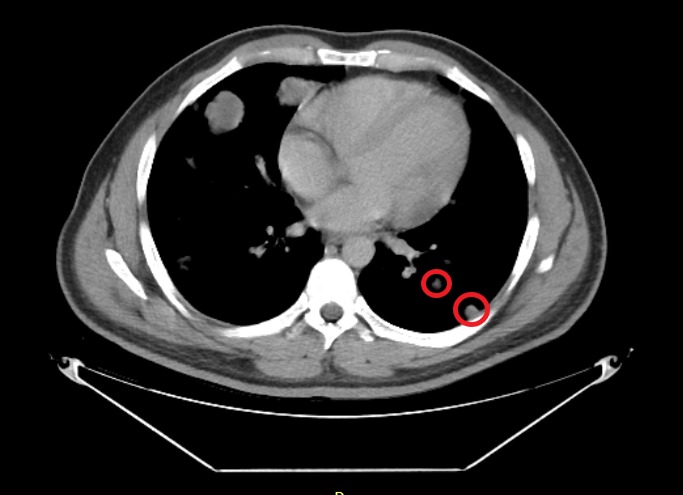
Chest computed tomography (CT) scan showing multiple metastatic pulmonary lesions (circles) on the left side

**Figure 7:**
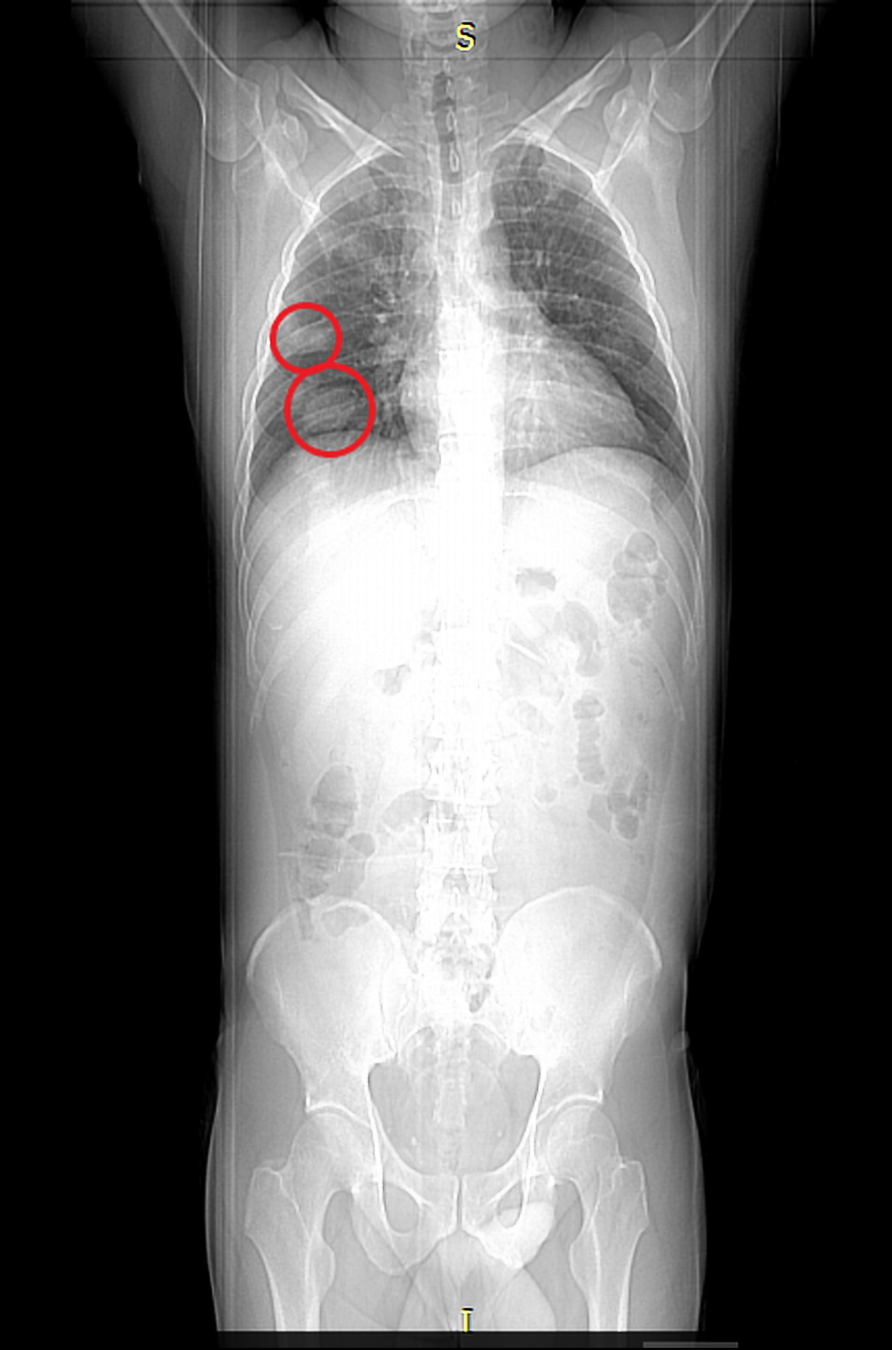
Scan projection radiograph of the anterior chest showing pulmonary nodules (red circles)

**Figure 8:**
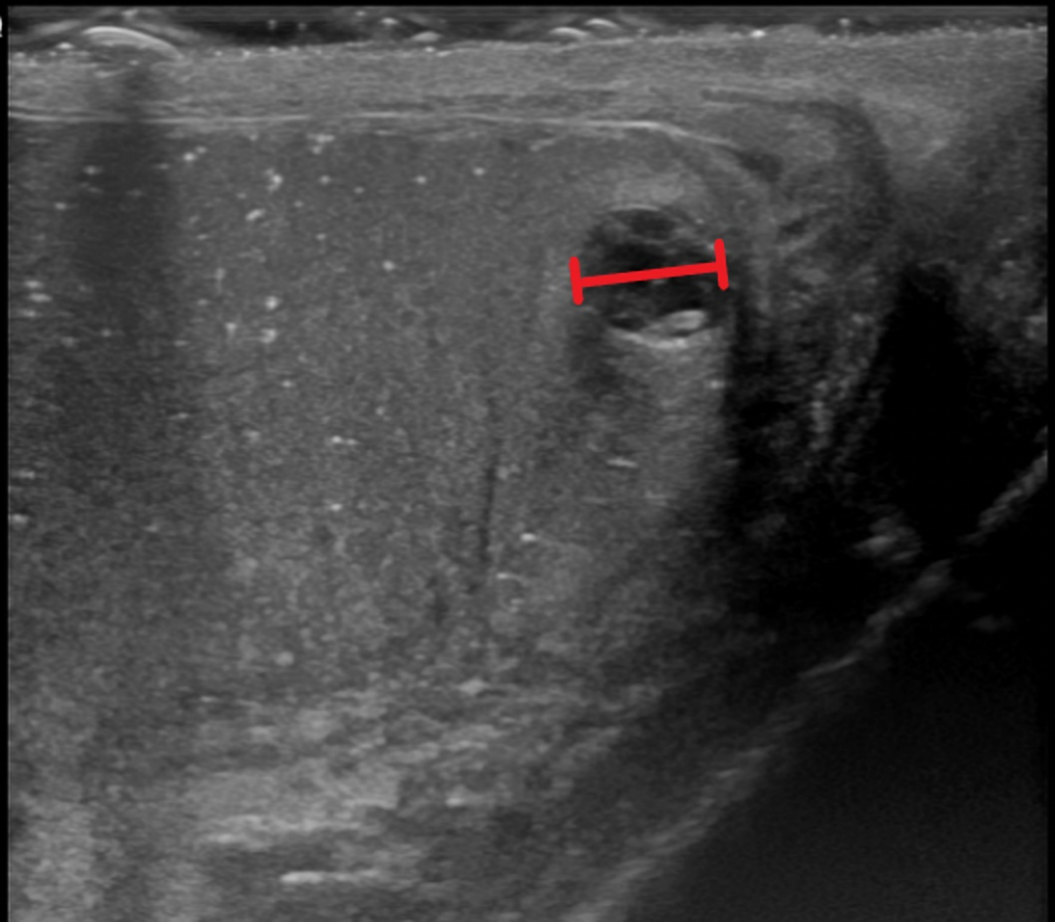
Ultrasound of the right testicle showing a complex multi-septated mixed lesion (red line)

**Figure 9:**
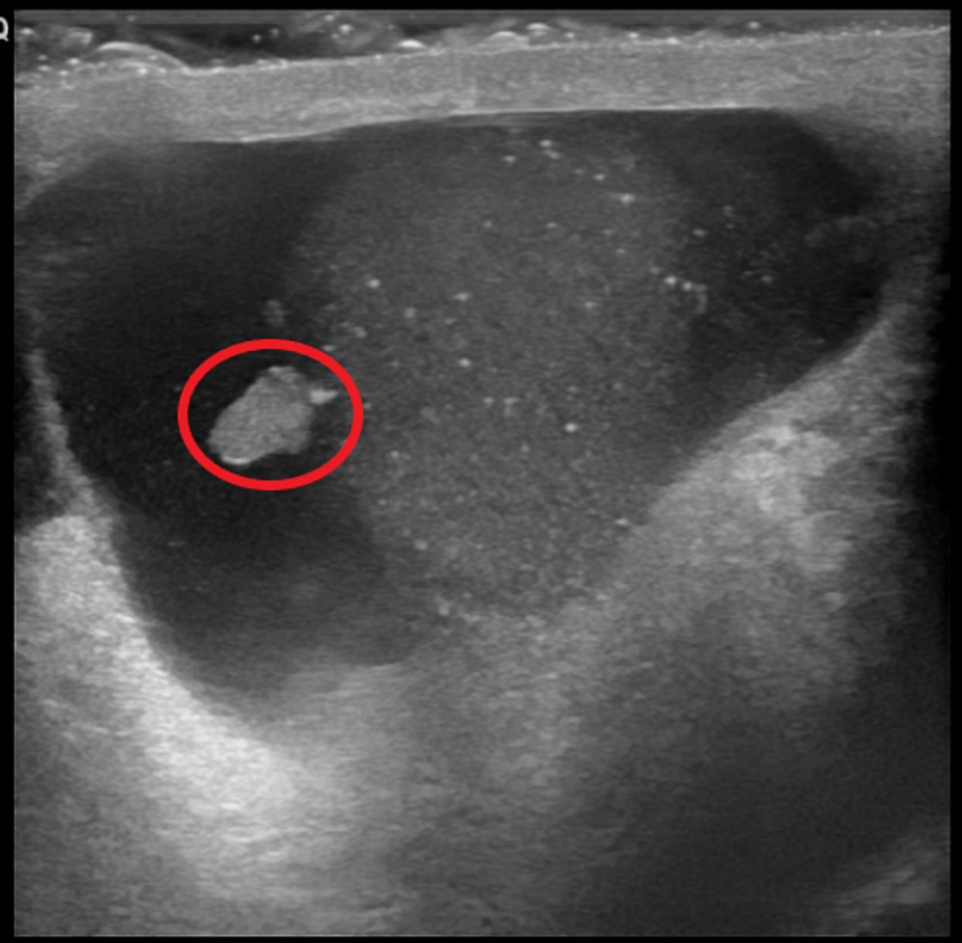
Ultrasound of the right testicle showing a hyperechoic mass on the inferior pole of the right testicle (red circle)

Computed tomography (CT) scan of the abdomen showed low-density lesions in the liver (Figures 1-2). Pelvic CT scan showed a right adrenal mass (Figure 3). A large retroperitoneal mass with some necrosis was seen with anterior displacement of the aorta (Figure 4). CT scan of the chest showed multiple pulmonary metastatic lesions (Figures 5-6). An anterior projection scan also showed multiple pulmonary lesions (Figure 7). An inferior ultrasound of the right testicle showed a complex multi-septated mixed lesion confined to the inferior pole of the right testicle (Figure 8). A superior view of the right testicle showed a mass with varying echogenicity (Figure 9). Measurements of the right testicle were 4.5 x 3.7 x 3.0 centimeters (cm) and 3.2 x 4.4 x 2.6 cm of the left testicle. There was no evidence of torsion. 

Hematology and oncology consultations were requested. Upon orchiectomy of the right testicle, cross sections were obtained and analyzed. The predominant component was consistent with a teratoma, and a small component was present with embryonal carcinoma. Immuno-staining for CD30, CD117, and OCT4 were performed to confirm this. However, the focus was no longer present in the tissue block. The morphological findings were still compatible with a small focus of embryonal carcinoma. Clinically, the patient was in a poor risk category with Stage IIIC disease. He was given a combination of cisplatin, etoposide, and bleomycin every three weeks for four cycles.

Biopsy Results

Upon orchiectomy, the tumor was very small in size and difficult to visualize on gross inspection. The tumor was unifocal and its macroscopic extent was confined to the inferior pole of the right testicle. The lesion was 1 cm in diameter and less than 3 millimeters (mm) for the embryonal carcinoma component. The surgical resection margin and tunica were free of any tumor. The microscopic tumor extension was limited to the testicular parenchyma only. Vascular invasion of the surrounding lymph nodes was not identified.

**Figure 10:**
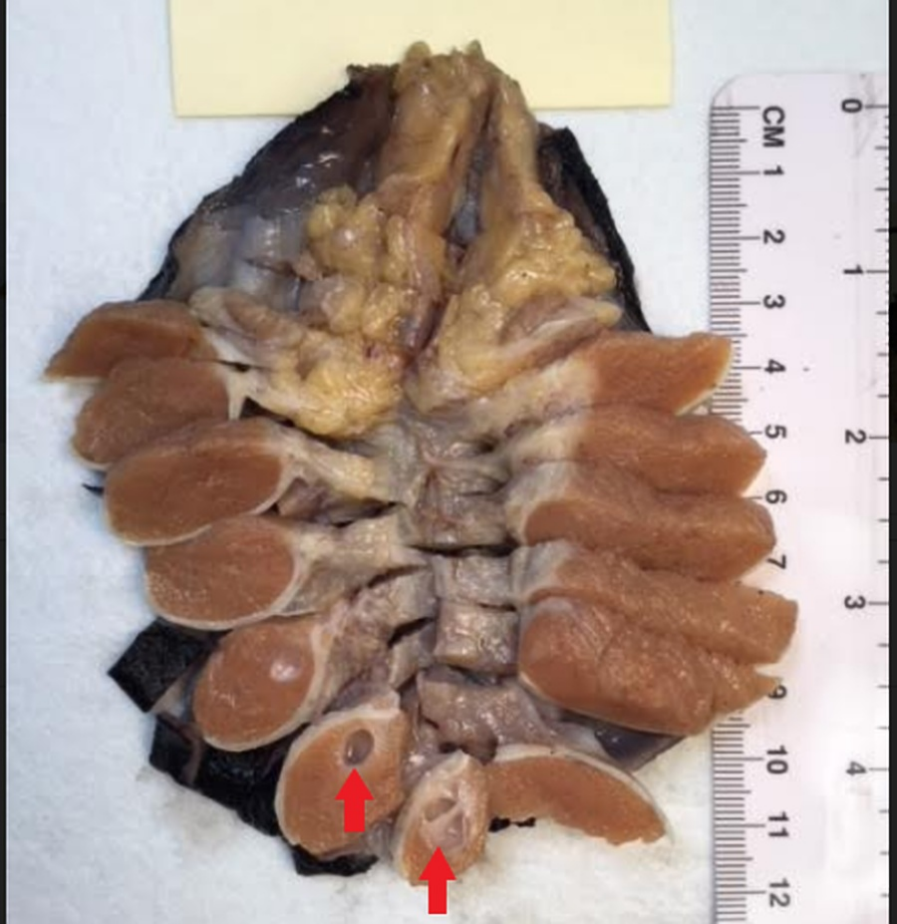
Cross-section of the right testicle showing cystic lesions on the inferior pole (red arrows)

A cross-section of the right testicle is shown in Figure 10. There was an accumulation of clear fluid between the tunica layers with approximately 10 milliliters (mL) of fluid. The attached epididymis was grossly unremarkable. Sections of the testicle revealed tan, soft parenchyma. The cystic fluid was clear and transparent. The inner surfaces were also smooth with no nodularity or papillary outgrowth. The tumor was well-defined from the surrounding tan testicular parenchyma. No other lesions were identified.

**Figure 11:**
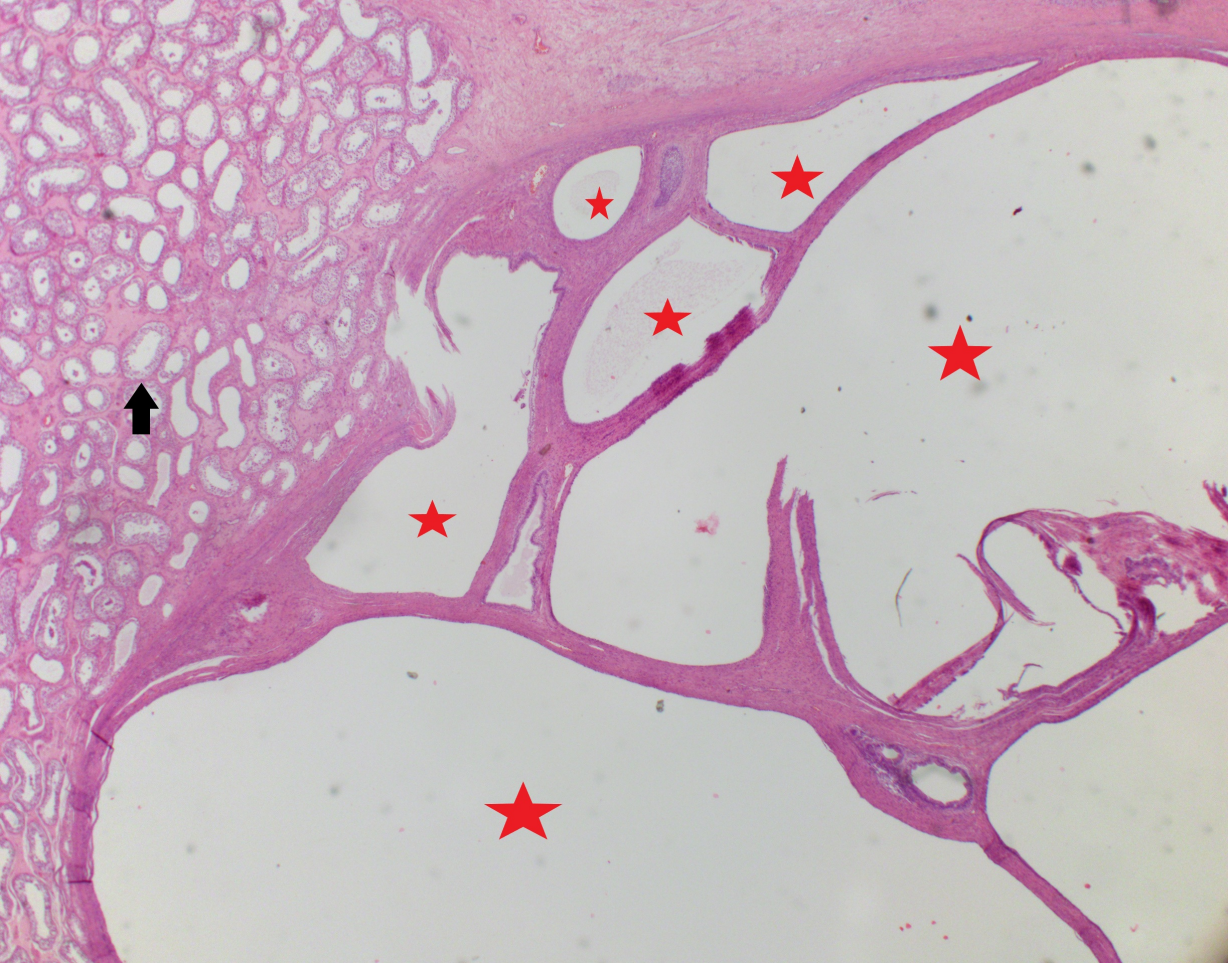
Multiloculated cysts (red stars) varying in size and shape Black arrow: seminiferous tubule

**Figure 12:**
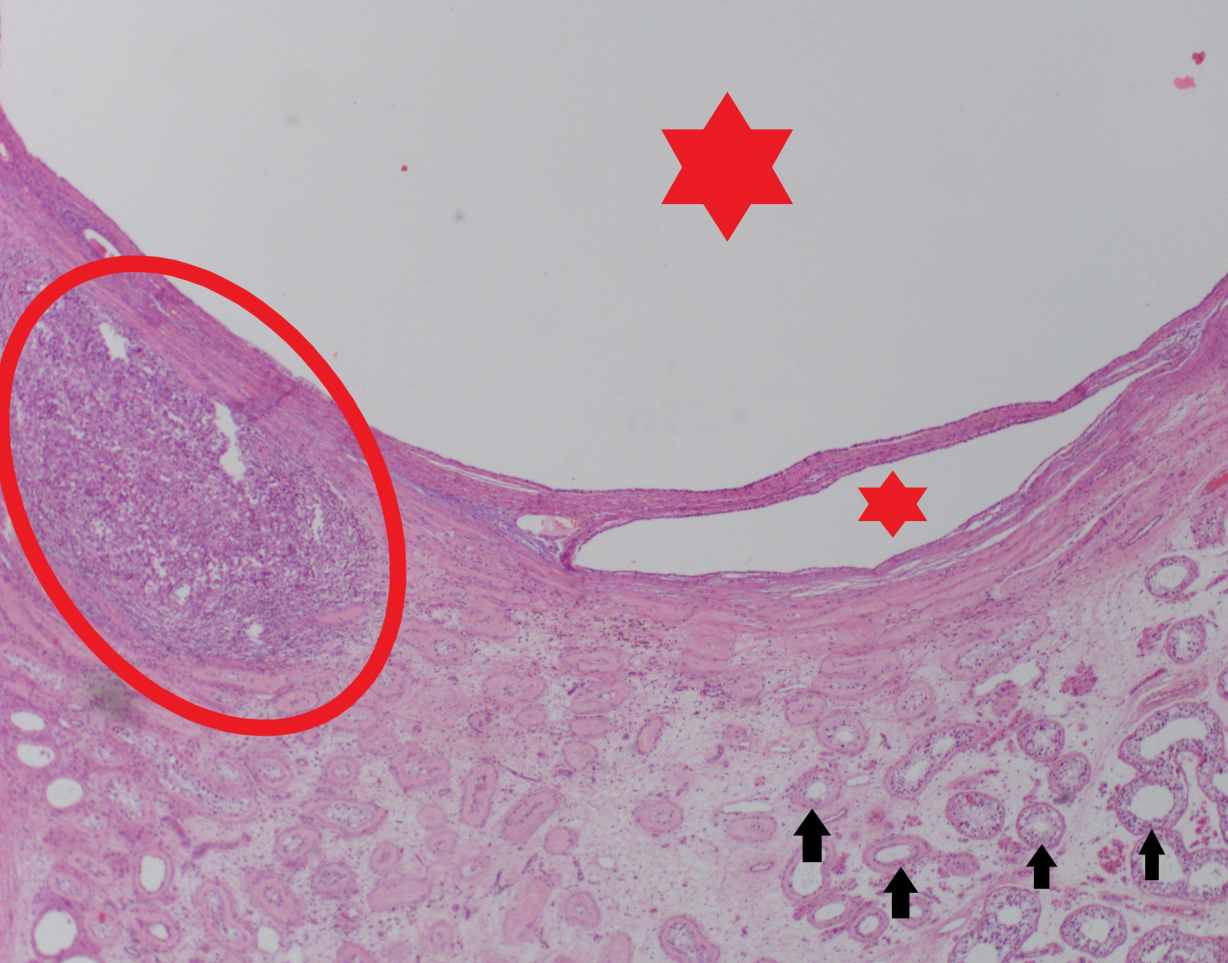
Cystic lesions (red stars) with a malignant focus located inferiorly (red circle) Black arrow: seminiferous tubules

**Figure 13:**
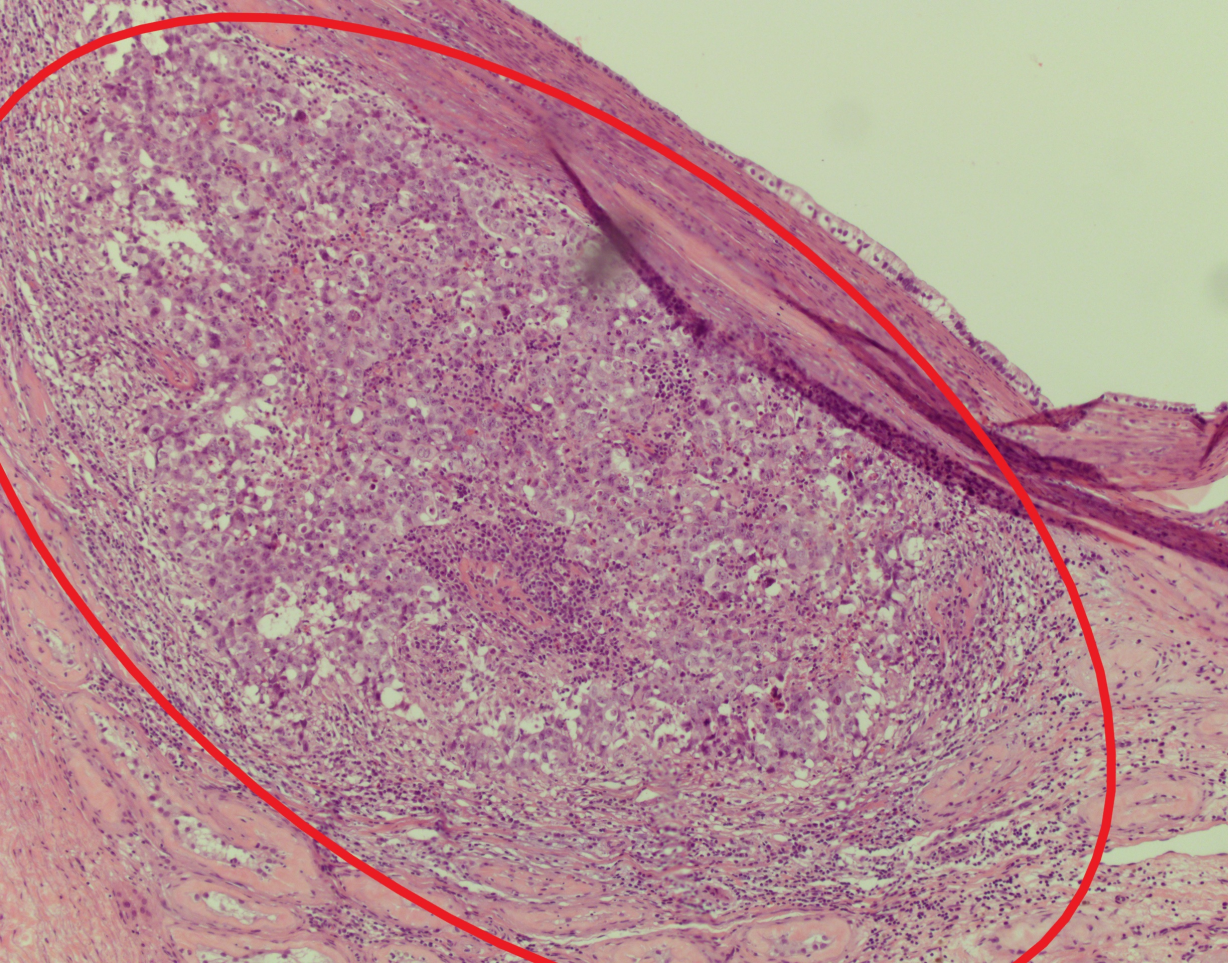
Medium-power focus of the malignant lesion (red circle)

**Figure 14:**
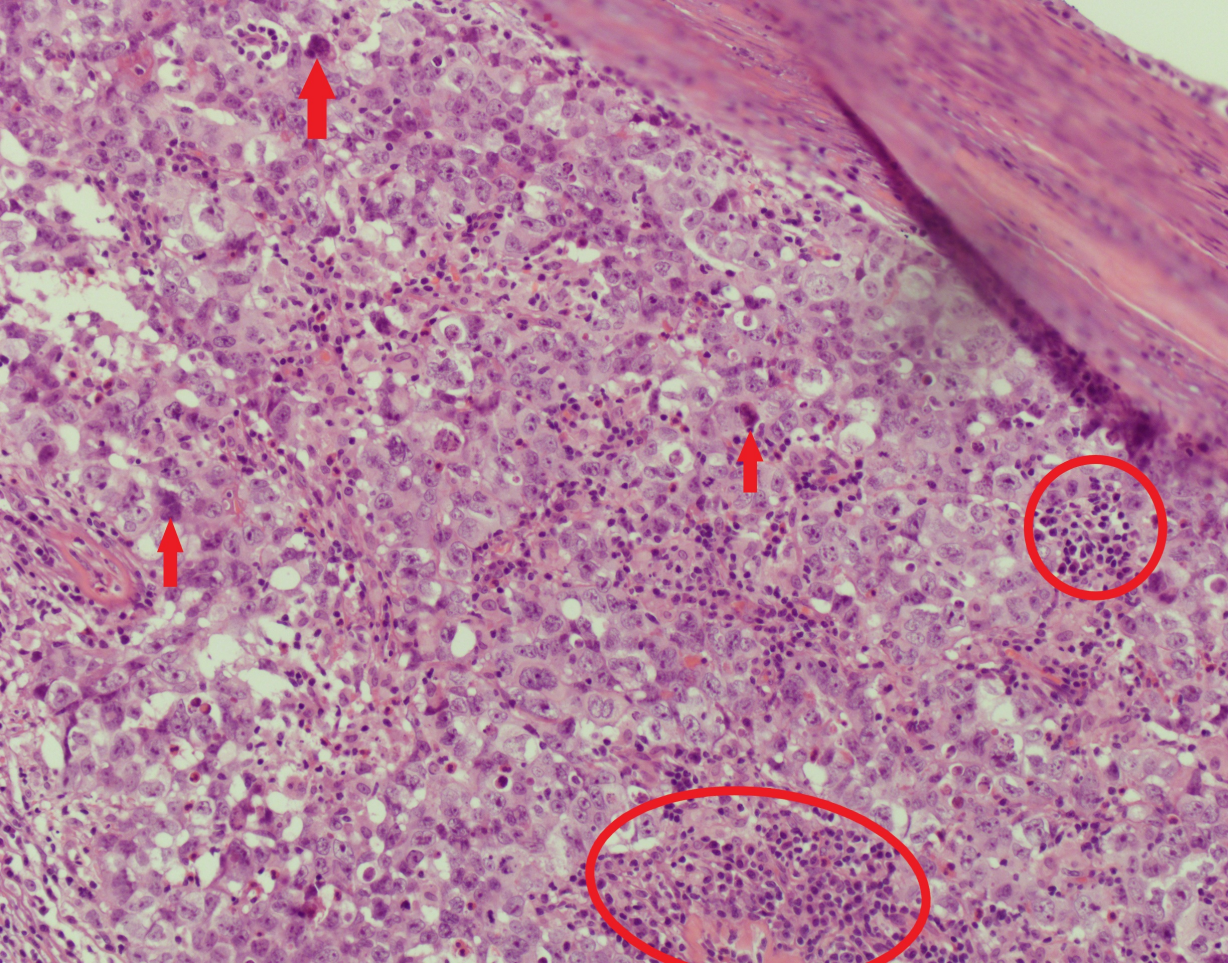
High-power focus of the malignant lesion, showing abundant nuclei (red circles) and prominent nucleoli (red arrows), with granular and eosinophilic cytoplasm

**Figure 15:**
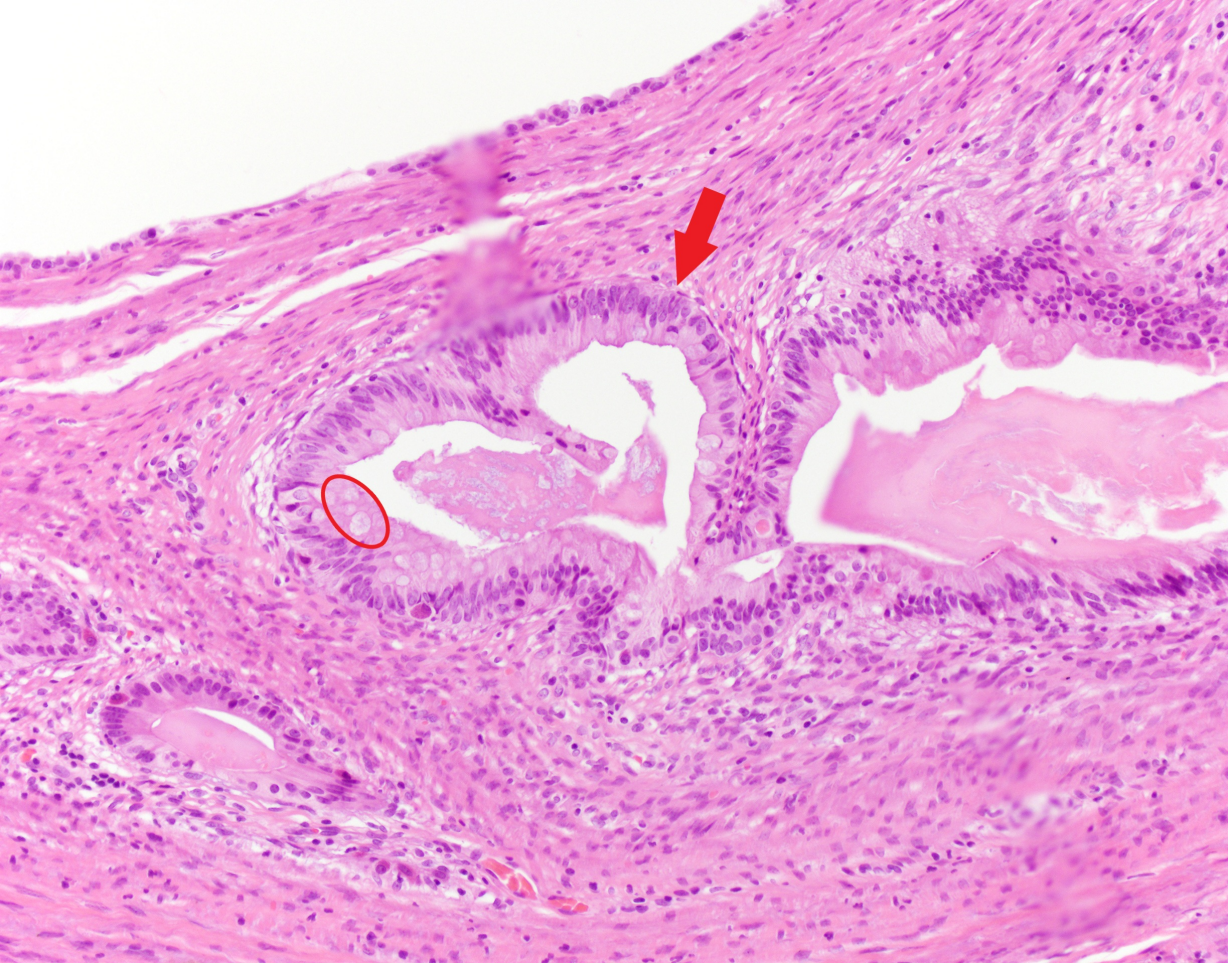
Cystic lesion lined by tall columnar epithelium (red arrow) with goblet cells (red circle)

**Figure 16:**
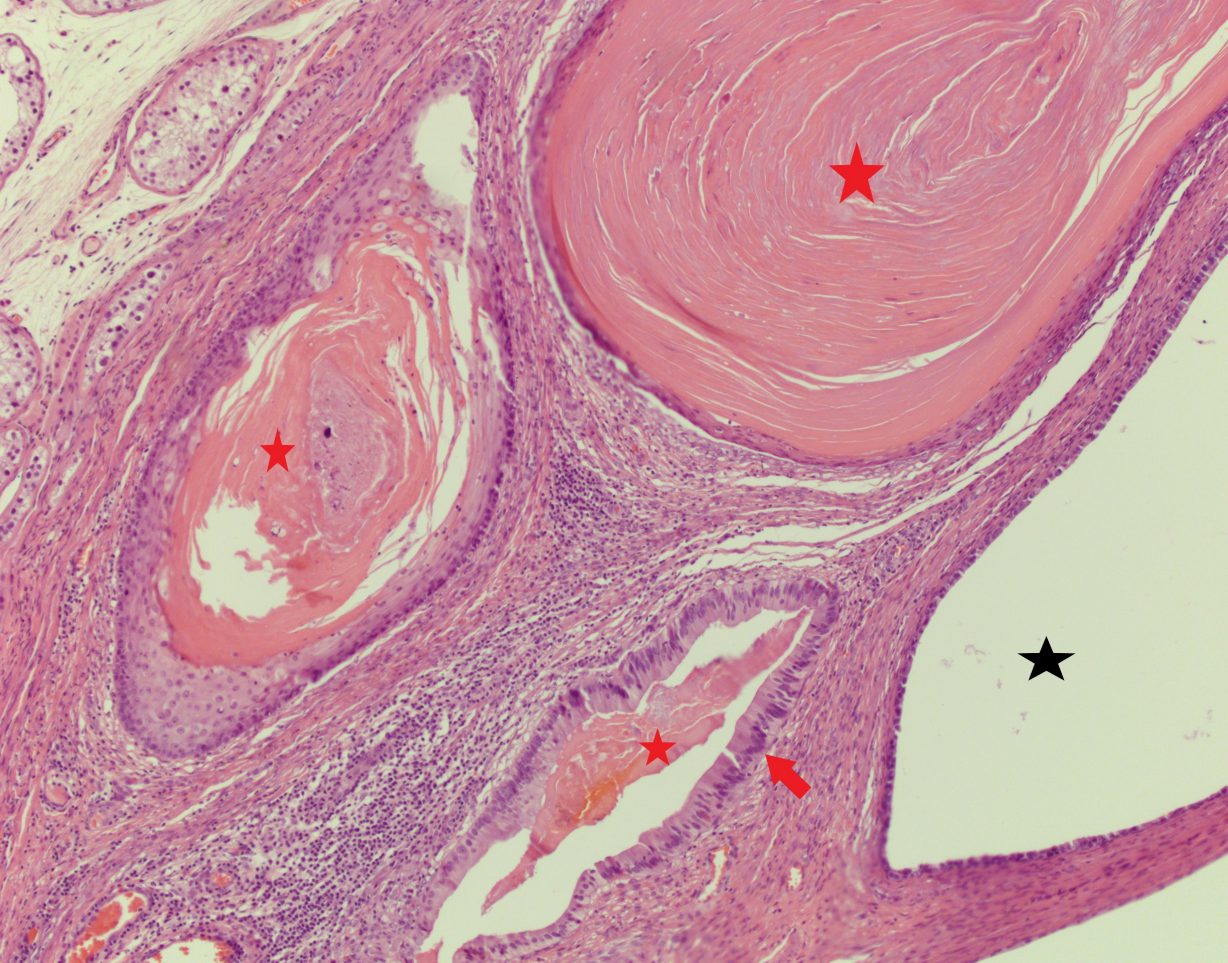
Concentric keratinization (red stars) confined within the cystic lesions, lined by tall columnar epithelium (red arrow) Black star: cystic lesion

Histological images are shown below in Figures 11-16. Histology confirmed multiloculated cystic lesions with small and large spaces. Some of these spaces were collapsed and compressed, and most were lined by tall columnar epithelium with goblet cells, characteristic of respiratory epithelium. A few small cystic areas and solid nests of squamous epithelium with keratinization were noted. A single focus was present within the fibrous tissue surrounding the cystic spaces. This focus was composed of large, highly atypical cells with enlarged irregular nuclei, prominent nucleoli, and atypical mitosis. There was a fair amount of granular and eosinophilic cytoplasm noted within the focus.

## Discussion

A pure teratoma in a male is very rare, and about one-third of cases are mixed germ cell tumors. Mature teratomas in post-pubertal males look like solid testicular tumors macroscopically. A disorganized arrangement of cells is seen microscopically. The pathogenesis of mixed testicular teratomas has been proposed to involve malignant germ cells that form non-teratoma elements first, which subsequently differentiate into teratoma elements [2]. Exhausted germ cell tumors can be seen in patients with metastatic testicular tumors with no gross evidence of the tumor [3]. This was most likely the reason why the gross tumor in our patient was barely seen upon orchiectomy.

In contrast to a mature teratoma, diagnosing an immature teratoma requires recognizing tissues that resemble cells of embryonic origin, specifically the presence of immature neural elements. An overgrowth of immature neural elements in teratomas is indicative of primitive neural ectodermal tumors (PNET) [2]. Metastatic spread of PNETs from germ cell tumors to areas outside the testicles have a high mortality rate and are often resistant to chemotherapy. However, the presence of PNETs within the testicle has not been shown to affect the prognosis [4]. Teratomas should not be categorized as either mature or immature in prepubertal or adult males because they carry a similar malignant potential [3].

Stratifying testicular cancer patients into having a good, intermediate, or poor prognosis is important when it comes to treatment [5]. The International Germ Cell Cancer Collaborative Group (IGCCCG) developed certain guidelines for staging patients with metastatic non-seminomatous germ cell tumors (NSGCT), based on the prognosis of patients after orchiectomy. These guidelines place an emphasis on tumor marker levels to categorize patients in the appropriate group. According to the IGCCCG, NSGCT’s with a good prognosis should meet all of the following criteria: The primary tumor should involve the testis or retroperitoneum, there should be no visceral metastasis to sites except the lungs, the alpha feto-protein (AFP) levels should be below 1,000 nanograms per milliliter (ng/ml), the human chorionic gonadotropin (hCG) levels should be below 5,000 international units per liter (IU/L), and the lactate dehydrogenase (LDH) levels should be below 1.5 times the upper limit of normal (ULN). NSGCT patients with an intermediate prognosis have criteria that are the same as a patient with good prognosis, except either the AFP level is between 1,000 to 10,000 ng/ml, the hCG level is between 5,000 to 50,000 IU/L, or the LDH level is between 1.5 to 10 x ULN. NSGCT patients with a poor prognosis should meet any one of the following criteria: The primary tumor is located in the mediastinum, there is visceral metastasis in sites outside the lungs, the AFP level is over 10,000 ng/ml, the hCG level is over 50,000 IU/I, or the LDH level is over 10 x ULN [5]. Based on the patient’s prognostic category, treatment should be tailored accordingly.

According to the IGCCCG risk classification, the treatment of choice for metastatic disease in NSGCT patients with a good prognosis is three cycles of the bleomycin, etoposide, and cisplatin (BEP) triple regimen. This triple regimen is superior to the cisplatin, vinblastine, and bleomycin (PVB) regimen in patients with advanced disease [5]. A five-day regimen is preferred over a three-day regimen because studies show that the three-day regimen is associated with a higher risk of toxicity [6]. In metastatic NSGCT with an intermediate prognosis, the treatment of choice is four cycles of the BEP regimen. In metastatic NSGCT with a poor prognosis, one cycle of BEP is recommended, followed by re-assessing the tumor markers after three weeks. If there is a favorable decline, BEP should continue for up to four cycles. If the decline in tumor markers is unfavorable, then a more intense chemotherapy regimen can be given [5].

Review of literature

There have been five reported cases of adult primary teratoma of the testes. All five cases were in clinical Stage I of the disease. Porcaro et al. retrospectively studied 75 patients from years 1976 to 2000 who had an orchiectomy for Stage I of NSGCT of the testis. Testicular teratoma was observed in five patients. All cases had elevated tumor markers. Four out of five patients underwent retroperitoneal lymph node dissection and one patient underwent surveillance. Two cases involved the right testicle and three cases involved the left testicle. Pure mature teratoma was seen in three out of five cases. Immature teratoma and teratoma with malignant transformation was seen in one out of five cases. A quarter of the patients had metastatic disease with a focus on embryonal carcinoma detected in one dissected lymph node. All of the five patients were disease-free with no relapses during follow-up. They concluded that these patients should undergo a close and long-term follow-up because a metastatic pure teratoma can clinically develop after 10 or more years [7].

## Conclusions

To our knowledge, a metastatic mature teratoma with a focus of embryonal carcinoma in a male is rare. Tumors may be very small or barely visible upon orchiectomy in metastatic disease. The treatment plan and duration following orchiectomy should be tailored according to proper stratification of the patient’s prognosis. A better understanding of testicular germ cell tumors is needed to help improve the clinical management of patients with end-stage disease. 
